# Supplementary material for: Antiproliferative Illudalane Sesquiterpenes from the Marine Sediment Ascomycete Aspergillus oryzae
Source: Mar Drugs. 2021 Jun 10;19(6):333. doi: 10.3390/md19060333 (PMC8230370; doi:10.3390/md19060333)
Supplement: Supplementary file 1 [file marinedrugs-19-00333-s001.zip › marinedrugs-1248394-supplementary.pdf]

SUPPLEMENTARY MATERIAL

Antiproliferative Illudalane Sesquiterpenes from the Marine Sediment Ascomycete *Aspergillus oryzae*

Raha Orfali[1], Shagufta Perveen[1*], Muhamamd F. Khan[2], Atallah F. Ahmed[1], Areej Al-Taweel[1], Mohammad A. M. Wadaan [2], Fahad A. Nasr[3], Sobia Tabassum[4], Paolo Luciano[5], Giuseppina Chianese[5], Jyh-Horng Sheu[6], and Orazio Taglialatela-Scafati[5,*]

1Department of Pharmacognosy, College of Pharmacy, King Saud University. P. O. Box 22452, Riyadh 11495, Kingdom of Saudi Arabia, rorfali@ksu.edu.sa, amaltaweel@ksu.edu.sa, alalqahtani@ksu.edu.sa

2Bio-products research chair, Department of Zoology, College of Science, King Saud University, P.O. Box 2455, Riyadh 11451, Saudi Arabia, fmuhammad@ksu.edu.sa

3Medicinal, Aromatic and Poisonous Plants Research Center, College of Pharmacy, King Saud University, P.O. Box 2455, Riyadh 11451, Saudi Arabia. fnasr@ksu.edu.sa

4Interdisciplinary Research Centre in Biomedical Materials (IRCBM), COMSATS University Islamabad, Lahore Campus, Pakistan; sobiatabassum@cuilahore.edu.pk

5Department of Pharmacy, School of Medicine and Surgery, University of Naples Federico II, Via Montesano 49, 80131 Naples, Italy; pluciano@unina.it, g.chianese@unina.it

6Department of marine biotechnology and resources, National Sun Yat-sen university, kaohsiung 804, Taiwan; sheu@mail.nsysu.edu.tw

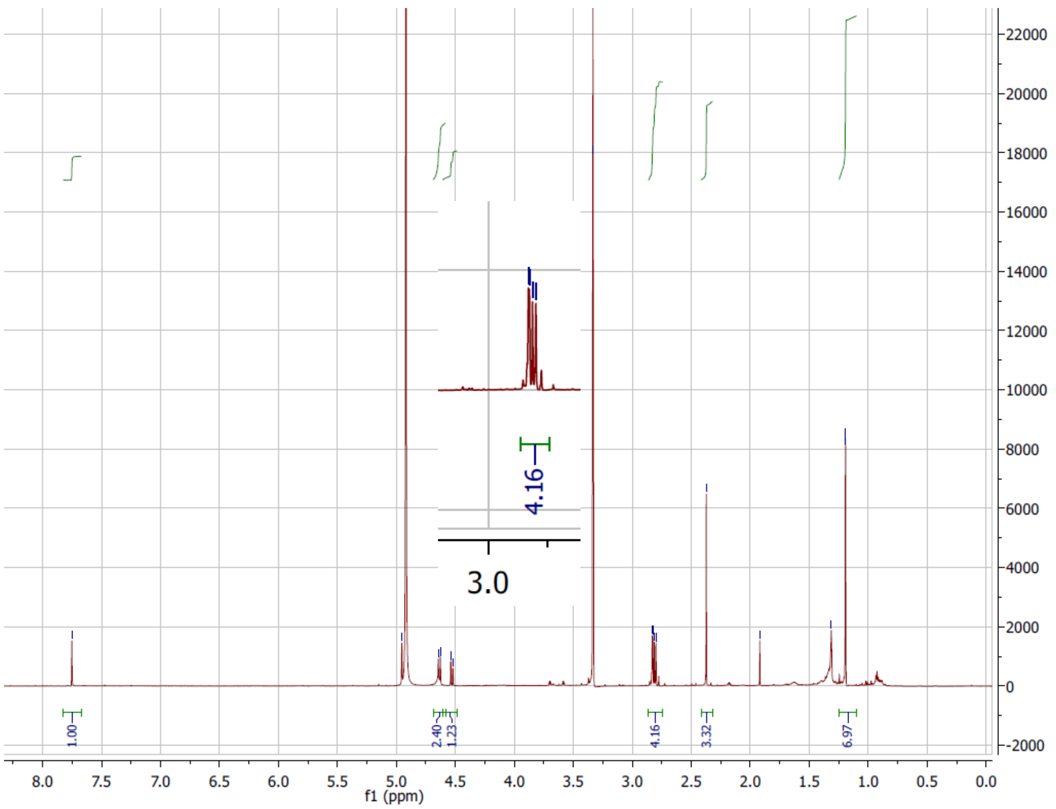

Figure S1. ^{1}H NMR (700 MHz) spectrum of asperorlactone in CD_3OD

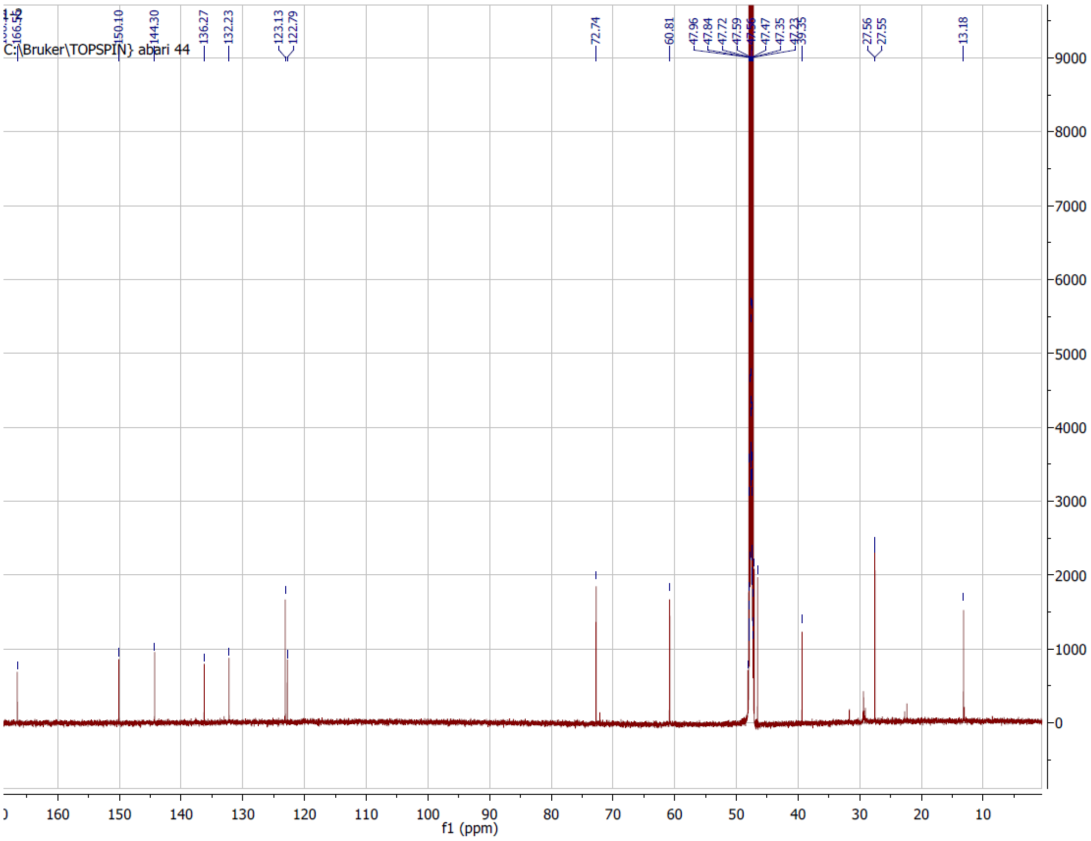

Figure S2. ^{13}C NMR (175 MHz) spectrum of asperorlactone in CD_3OD

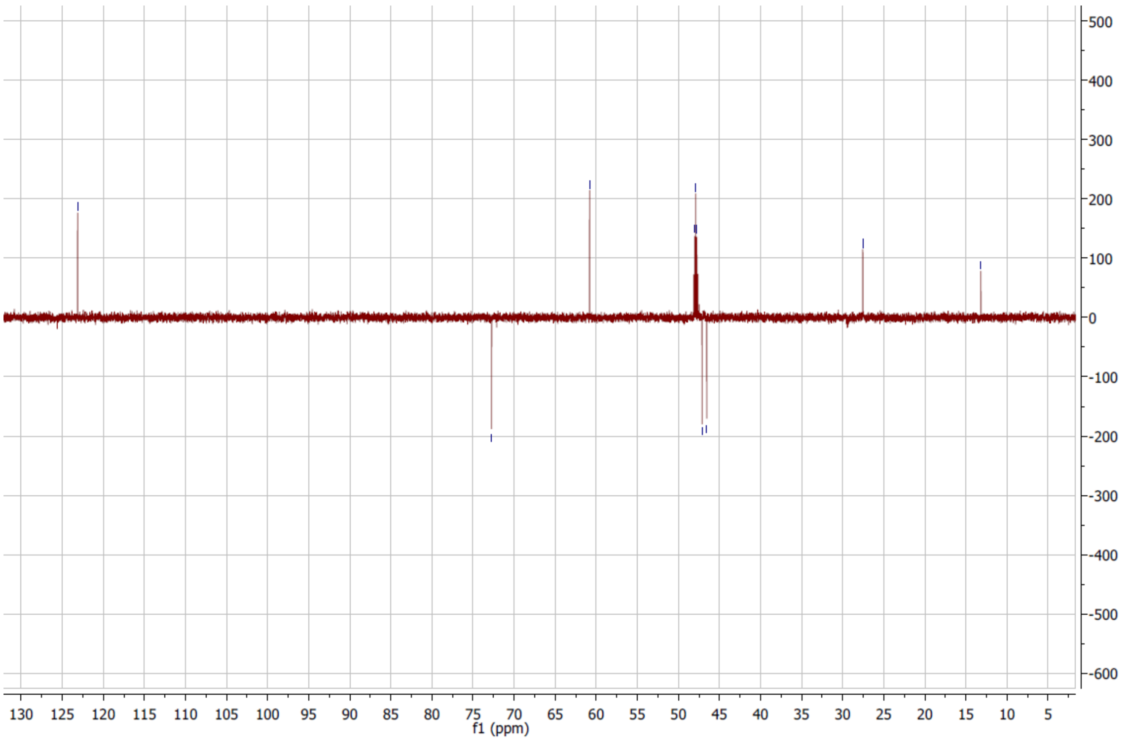

Figure S3. DEPT135 NMR (175 MHz) spectrum of asperorlactone in CD_3OD

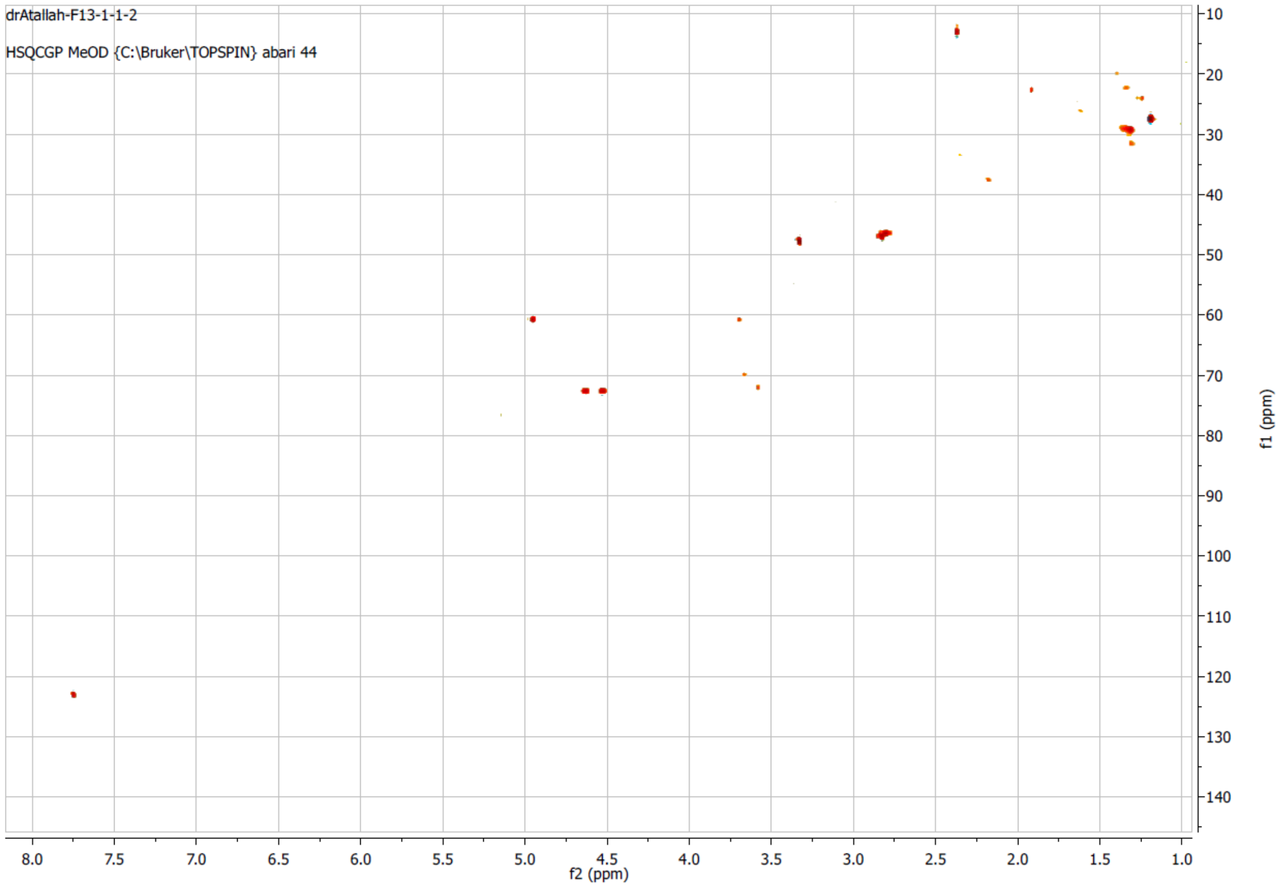

Figure S4. 2D NMR HSQC spectrum of asperorlactone in CD_3OD

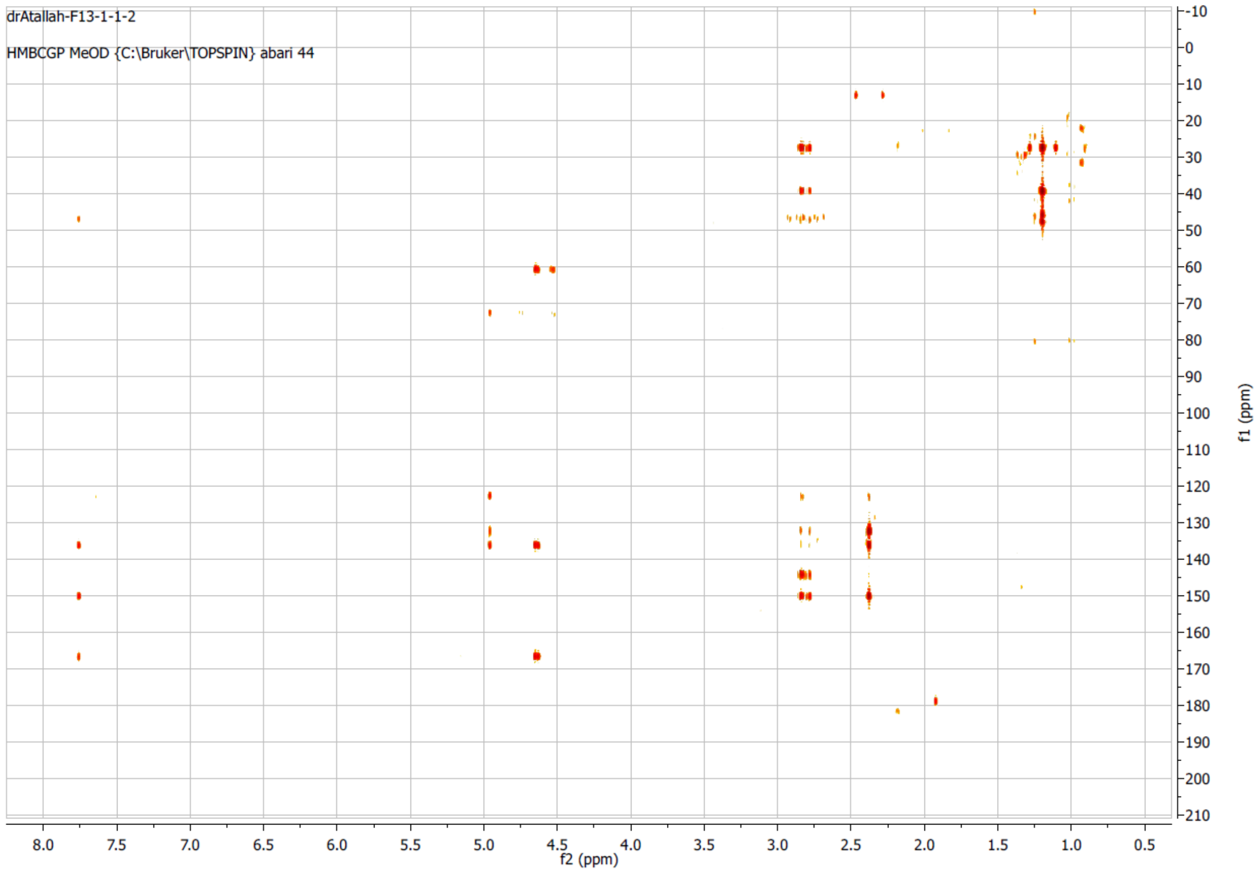

Figure S5. 2D NMR HMBC spectrum of asperorlactone in CD_3OD

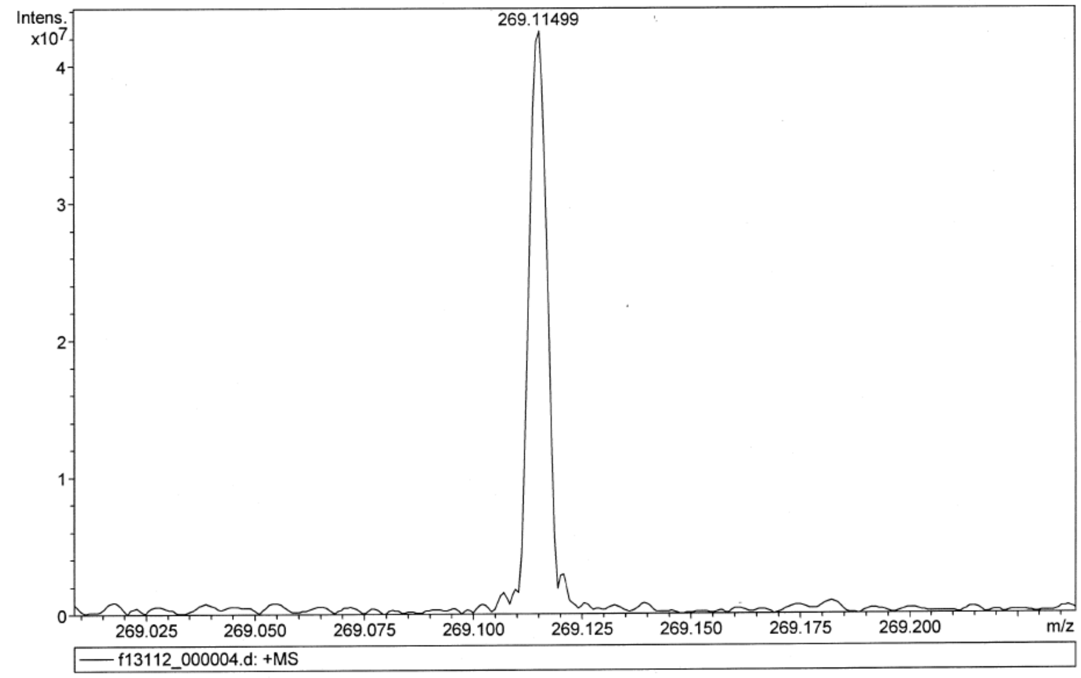

Meas. m/z	#	Formula	Score	m/z	err [mDa]	err [ppm]	mSigma	rdb	e⁻ Conf	N-Rule
269.11499	1	C 15 H 18 Na O 3	100.00	269.11482	-0.17	-0.65	10.7	6.5	even	ok

Figure S6. HR-MS spectrum of asperorlactone
